# Supplementary material for: Universal noninvasive prenatal diagnosis for monogenic disorders using cell-free plasma DNA
Source: Genome Med. 2025 Dec 4;18:4. doi: 10.1186/s13073-025-01588-5 (PMC12797552; doi:10.1186/s13073-025-01588-5)
Supplement: Supplementary file 1 — Additional file 1: Supporting fig S1-S13. Fig. S1. The workflow of this study. Fig. S2. Theoretical recall values for different variant frequencies and sequencing depths. Fig. S3. The sequencing depth for cell-free DNA and genomic DNA in this study. Fig. S4. Distribution of fetal fraction at different gestational ages in this study. Fig. S5. Maternal recombination in the GJB2 gene region affects the inference of fetal haplotypes in fam8. Fig. S6. Analysis of meiotic recombination events. Fig. S7. Number of informative SNPs for paternal and maternal haplotypes per 4-Mb genomic window. Fig. S8. CNV profiles for eight microdeletion/microduplication syndrome cases. Fig. S9. Quality-Controlframework for HaploNIPD. Fig. S10. Distribution of fetal fraction in samples collected between 8 and 12 weeks of gestation in our internal cohort. Fig. S11. Concordance of recombination breakpoints in our cohort with established hotspots. Fig. S12. Number of suitable SNPs in 1000 Genomes Project populations based on SNP selection criteria in this study. Fig. S13. Effect of SNP numbers on haplotype accuracy by downsampling simulation. [file 13073_2025_1588_MOESM1_ESM.docx]

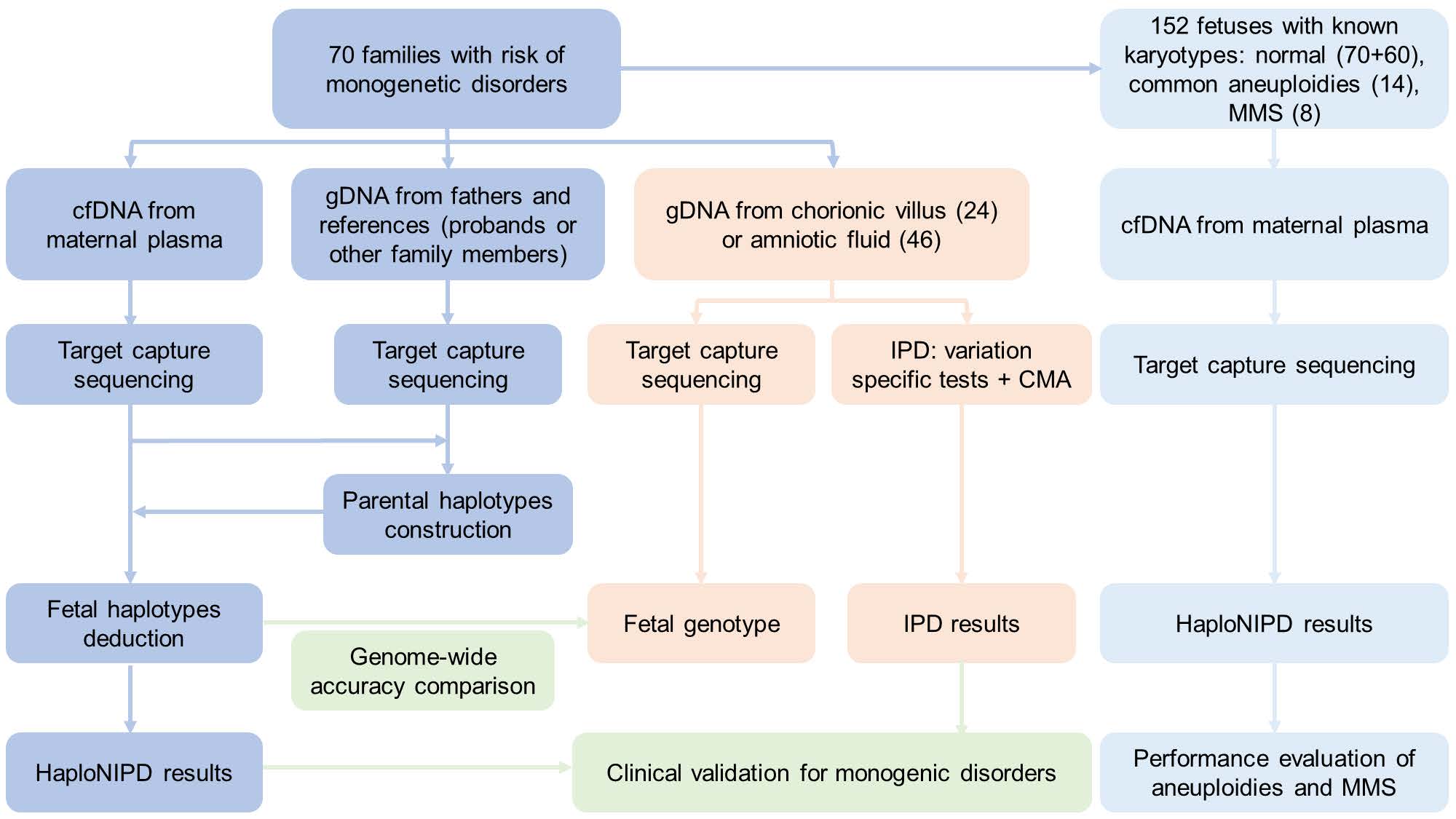
**Fig. S1. The workflow of this study.** Cell-free DNA (CfDNA) from maternal plasma and genomic DNA (gDNA) from fathers, probands or other family members (as reference) were subjected to target capture sequencing. Fetal genotypes were deducted through haplotype inheritance prediction. The accuracy of HaploNIPD were compared to those of invasive prenatal diagnosis (IPD). Additionally, target capture sequencing of fetal DNA from chorionic villus or amniotic fluid samples was performed for comprehensive genome-wide accuracy comparison. To assess aneuploidy and microdeletion/microduplication syndromes (MMS) detection capabilities, 152 maternal plasma samples with known fetal karyotypes confirmed by chromosomal microarray (CMA) were analyzed, including 14 aneuploid cases, 8 MMS cases, and 130 with normal karyotypes (including the 70 monogenic high-risk cases).


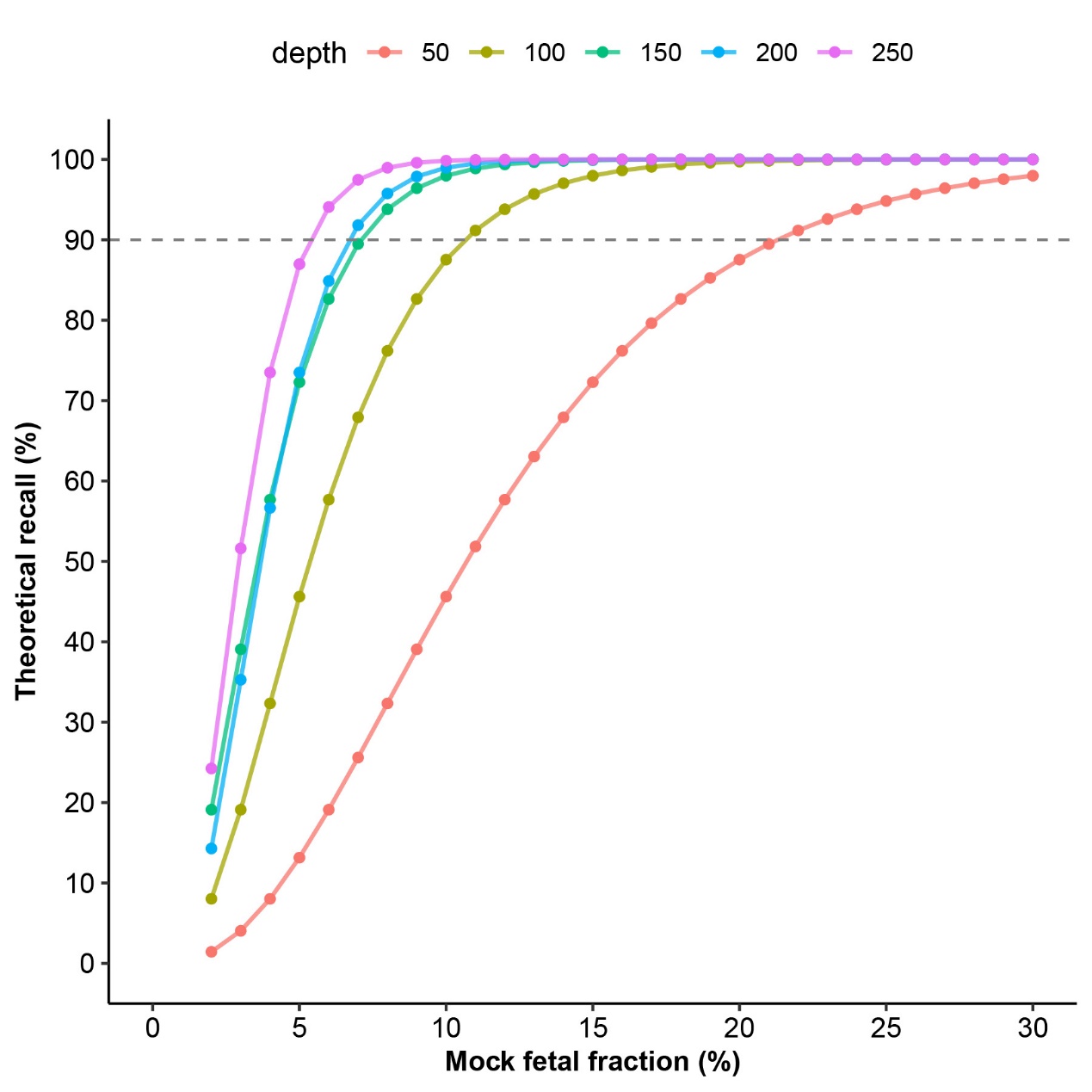


**Fig. S2. Theoretical recall values for different variant frequencies and sequencing depths**. Theoretical recall values for different combinations of variant frequency (half of the mock fetal fraction) and sequencing depth (50×, 100×, 150×, 200×, and 250×) were estimated using the Poisson cumulative density function.


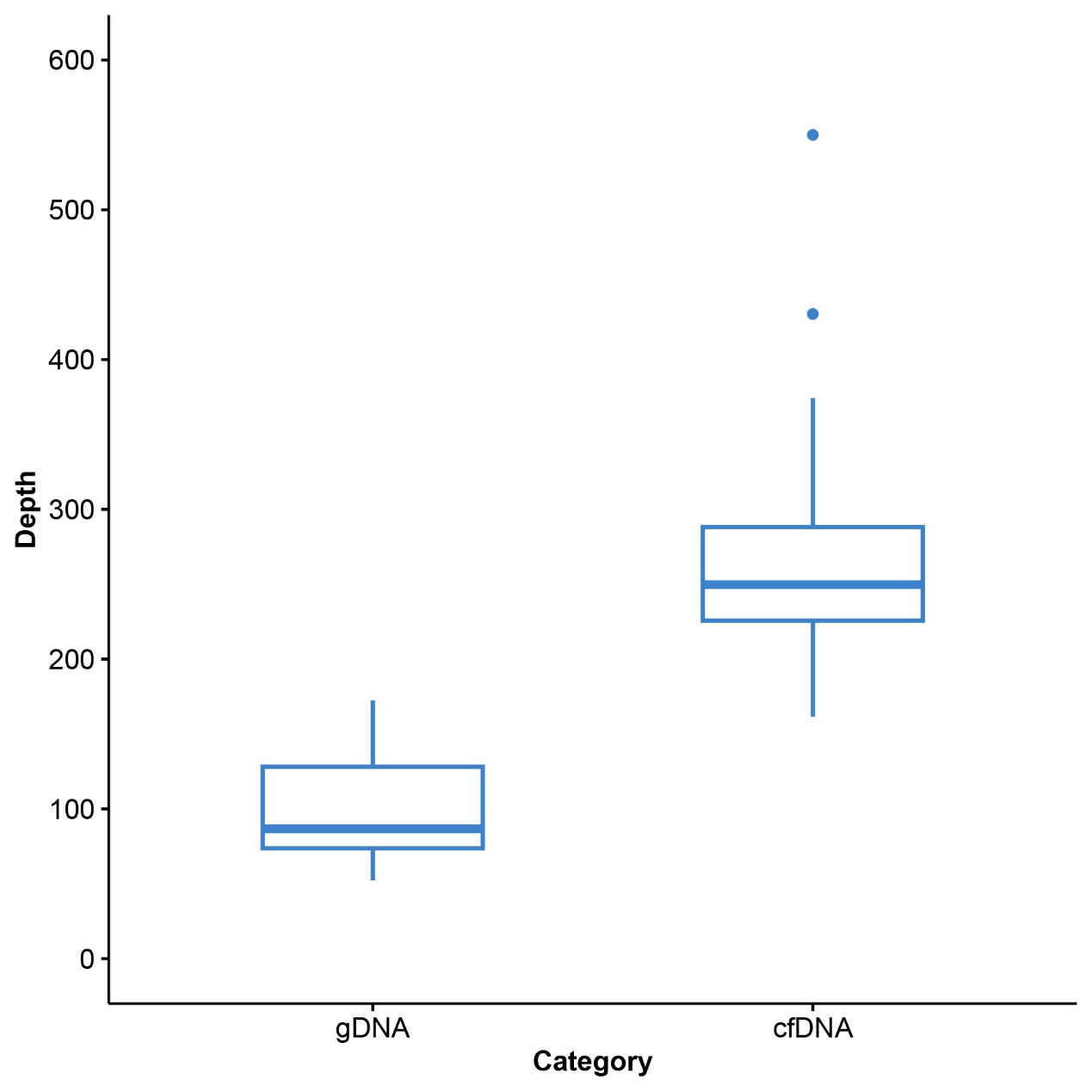


**Fig. S3**. **The sequencing depth for cell-free DNA (cfDNA) and genomic DNA (gDNA) in this study**. Boxplots show the median and interquartile range; mean values are reported in the main text.


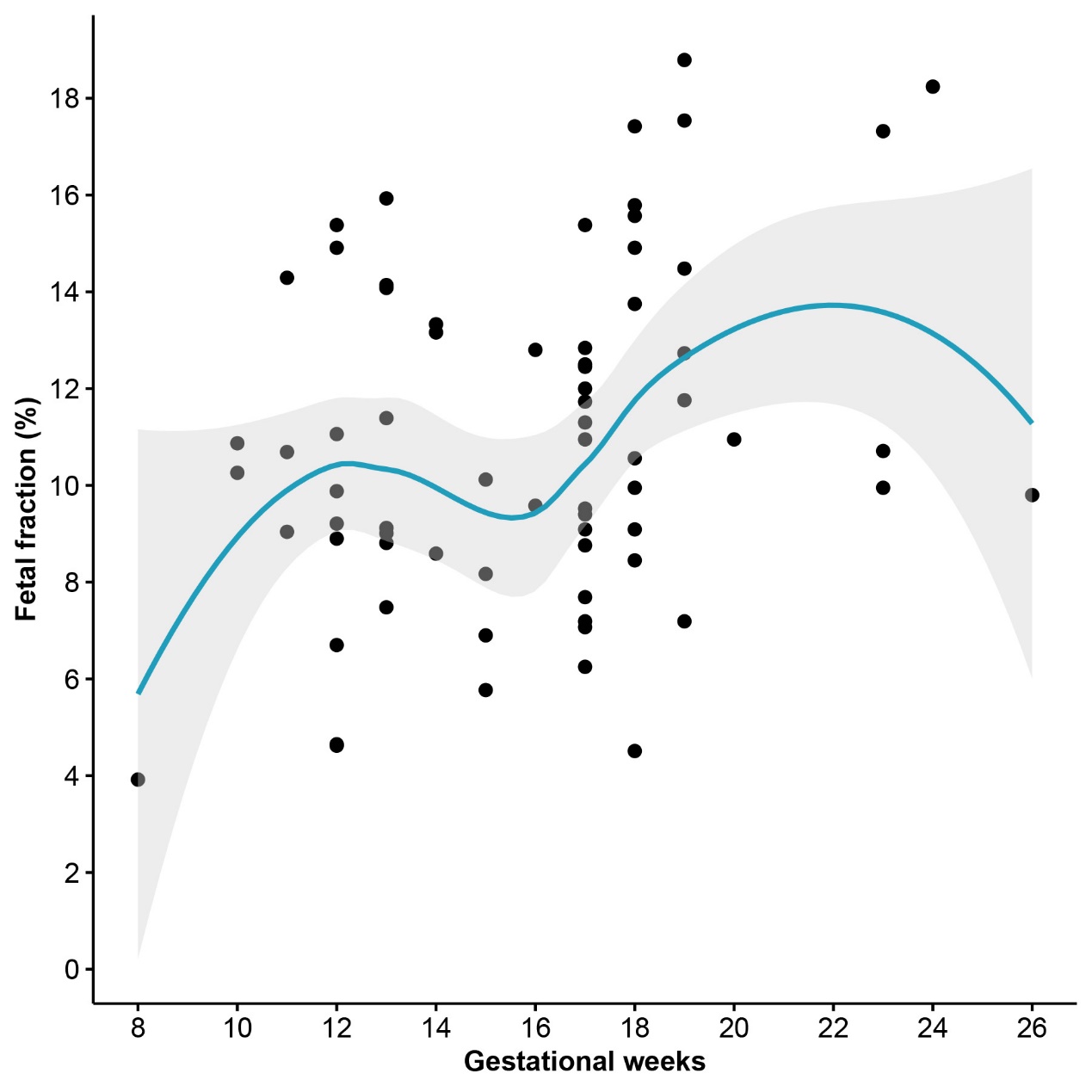


**Fig. S4**. **Distribution of fetal fraction at different gestational ages in this study.**


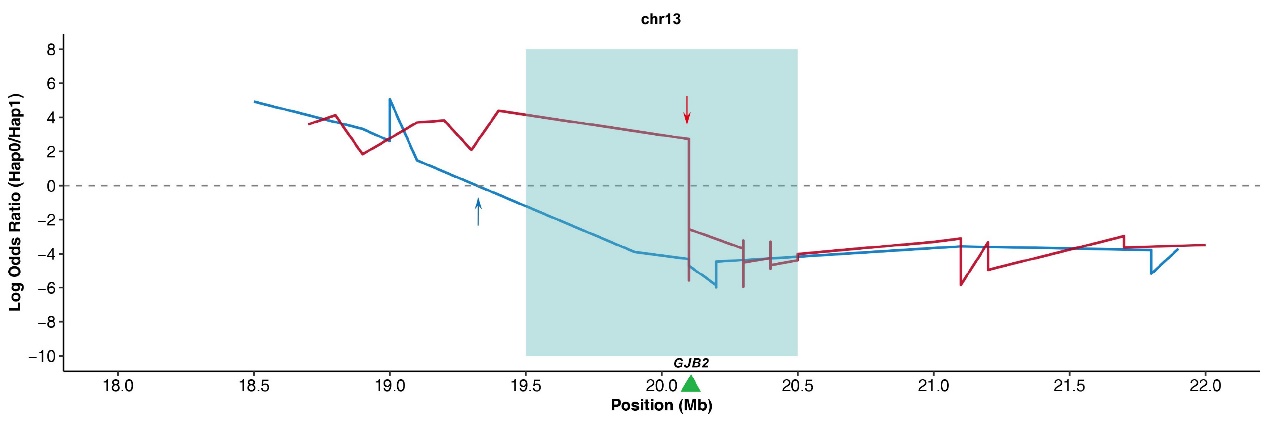


**Fig. S5**. **Maternal recombination in the *GJB2* gene region affects the inference of fetal haplotypes in fam8.** The x-axis represents the position on chromosome 13 nearby *GJB2* gene, while the y-axis represents the log odds ratios of fetal inheritance of paternal (blue line, Hap0 vs Hap1) and maternal (red line, Hap0 vs Hap1). Both parental haplotypes harbor recombination breakpoints in this region. The paternal breakpoint (~800 kb upstream of *GJB2*) encompasses 15 informative SNPs and does not affect inference of paternal transmission. However, the breakpoint in maternal haplotype is located in the genic region, resulting in an indeterminate outcome for the fetal inheritance of the maternal haplotype. Blue arrow, paternal breakpoint; red arrow, maternal breakpoint; shaded blue regions, targeted analysis intervals.

**
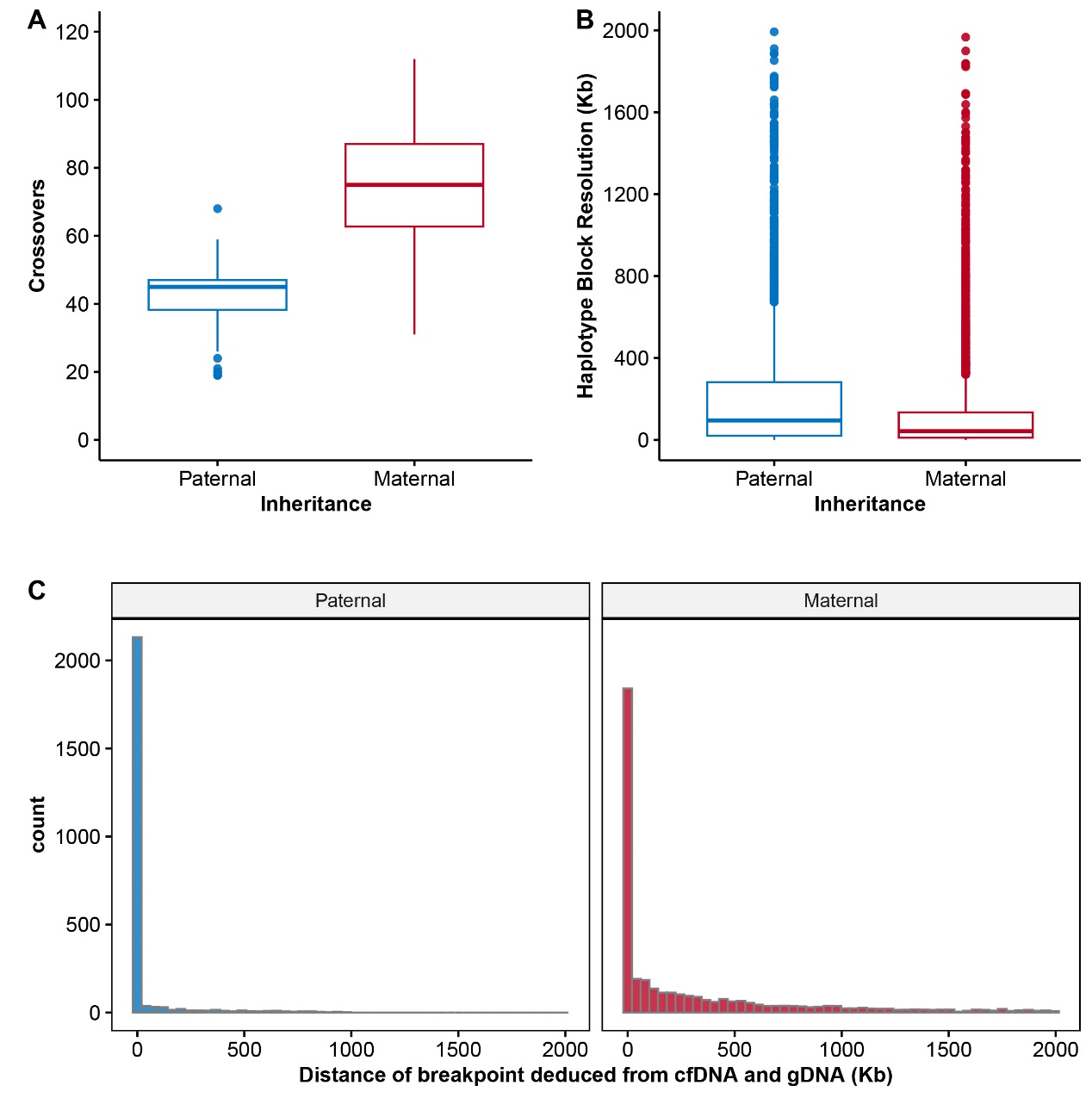
**

**Fig. S6. Analysis of meiotic recombination events. (A)** Distribution of paternal and maternal meiotic recombination events across samples. **(B)** Crossover resolution, defined as the distance between adjacent haplotype blocks, for paternal and maternal haplotypes. **(C)** Distances between crossover breakpoints inferred from cfDNA and those derived from fetal gDNA (reference standard) for both paternal and maternal events. CfDNA, cell-free DNA; gDNA, genomic DNA.

**
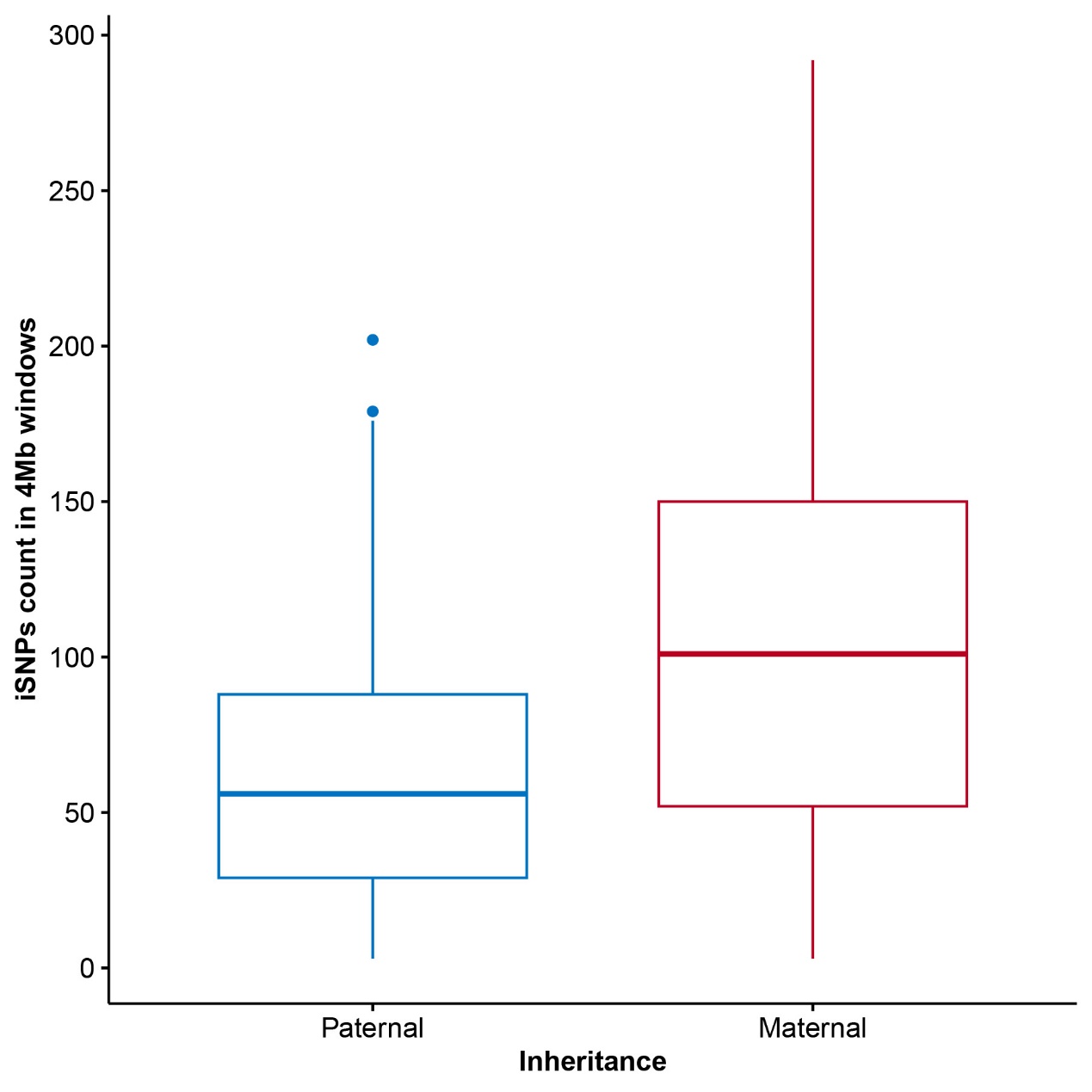
**

**Fig. S7. Number of informative SNPs (iSNPs) for paternal and maternal haplotypes per 4-Mb genomic window.**

**
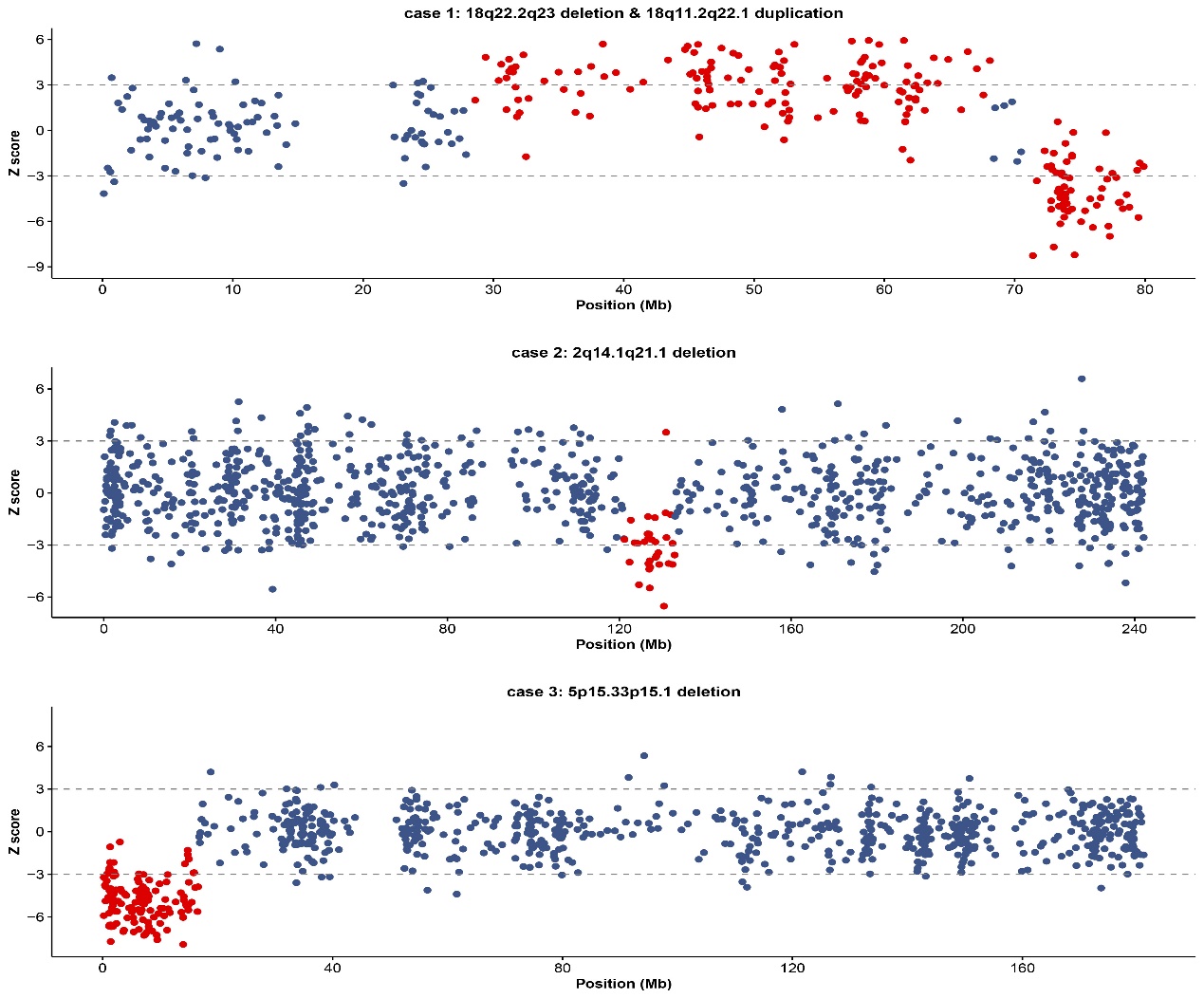

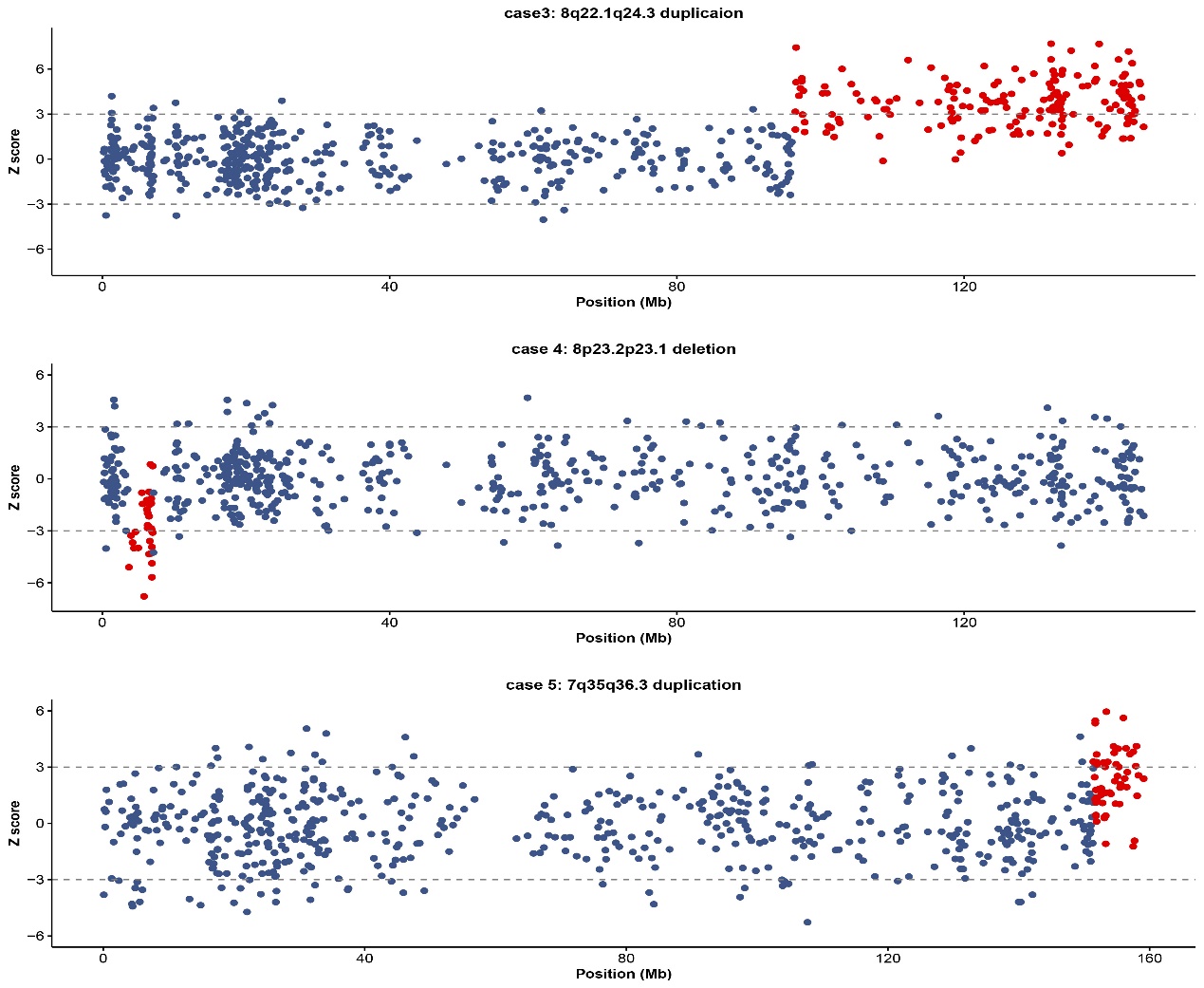

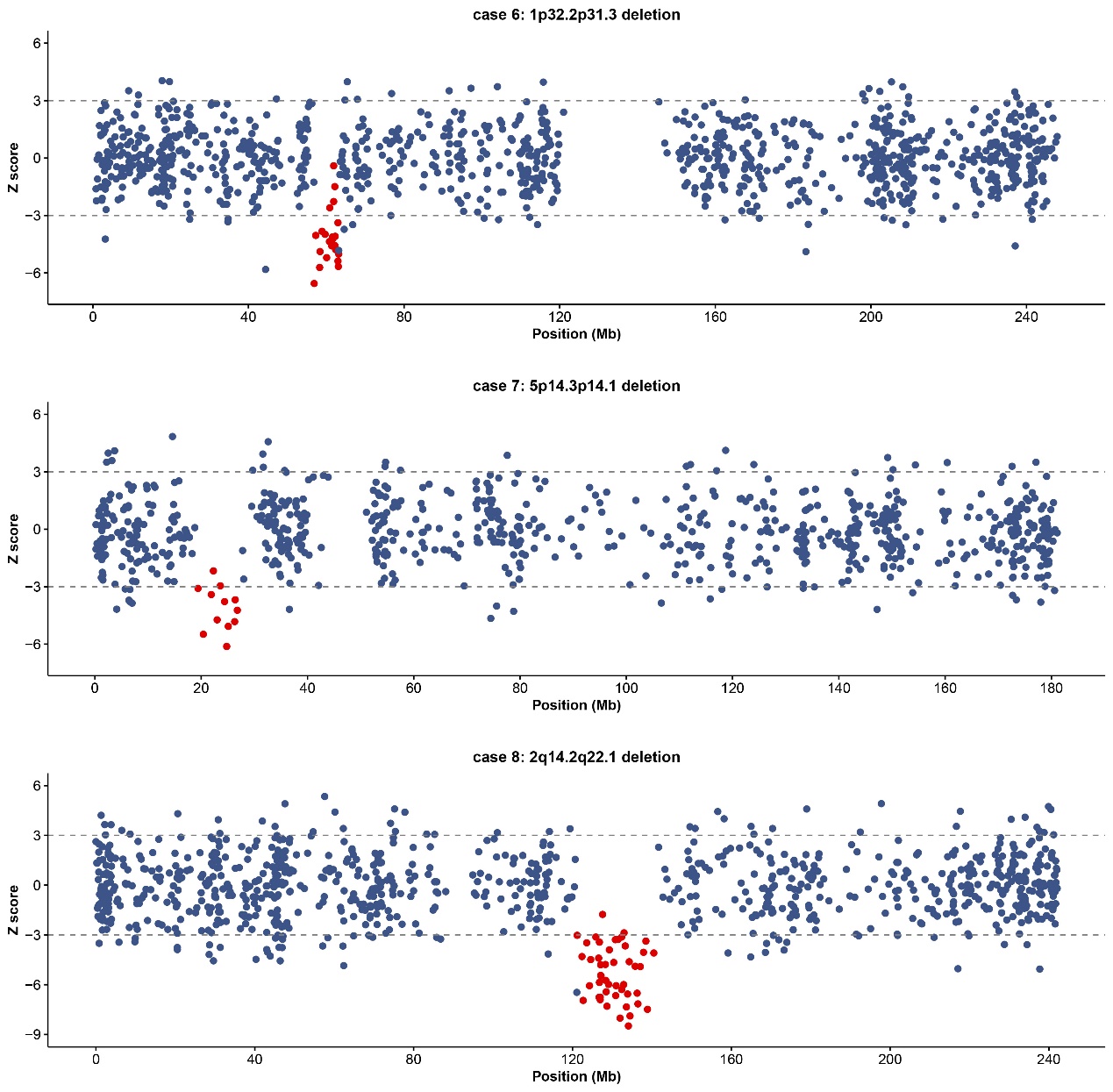
**

**Fig. S8.** **CNV profiles for eight microdeletion/microduplication syndrome cases.**

**
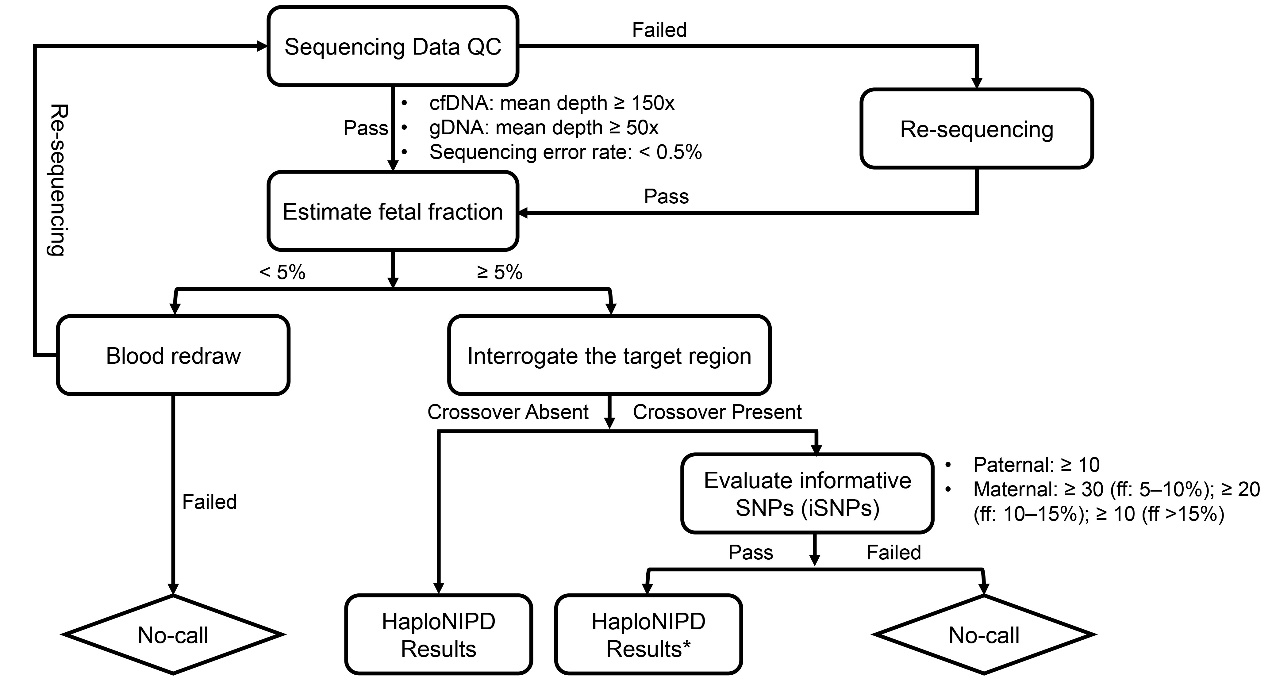
**

**Fig. S9. Quality-Control (QC) framework for HaploNIPD.** ff, fetal fraction. * indicate recombination event was observed near the target region and the result should be interpreted with caution.


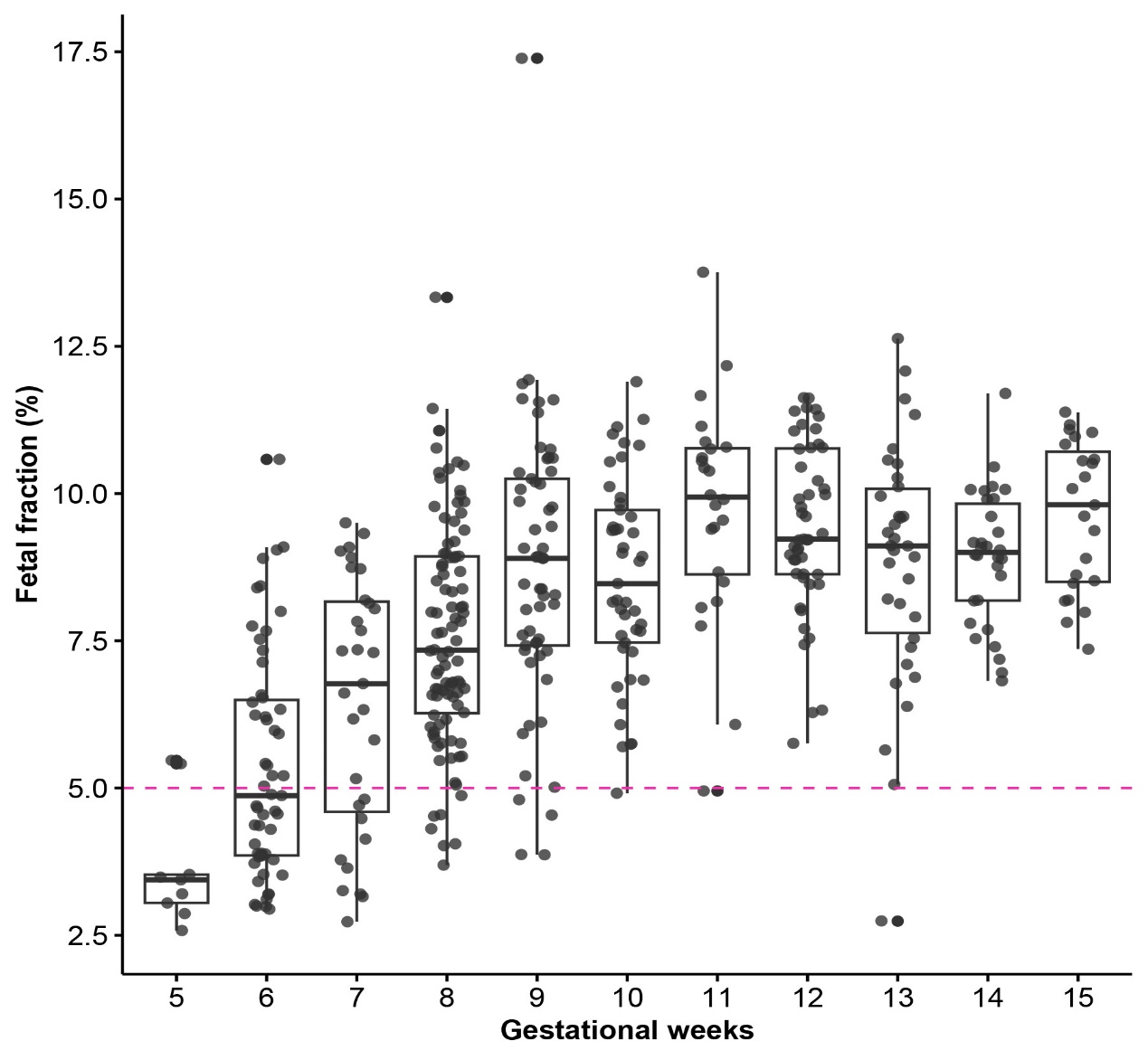


**Fig. S10. Distribution of fetal fraction in samples collected between 8 and 12 weeks of gestation in our internal cohort.**

**
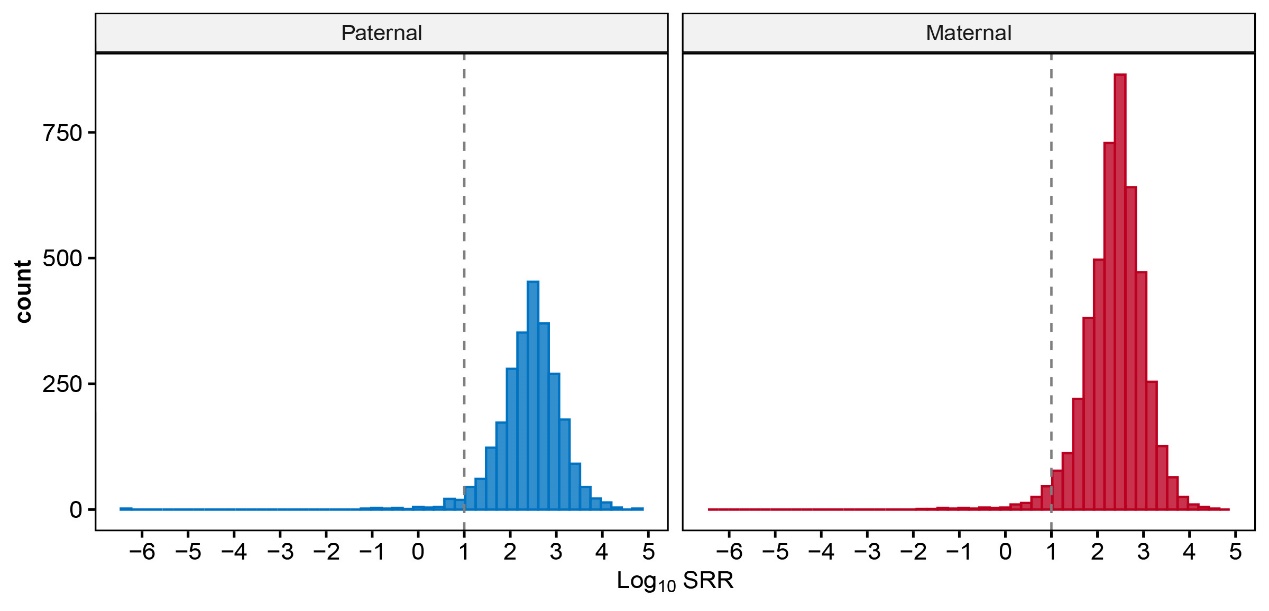
**

**Fig. S11. Concordance of recombination breakpoints in our cohort with established hotspots.** Distribution of the log₁₀-transformed standardized recombination rate (log_10_SRR) from the deCODE recombination map for genomic regions located within 1 Mb of paternal or maternal recombination breakpoints detected in our cohort. Values of log₁₀SRR ≥ 1 indicate localization within recombination hotspots.


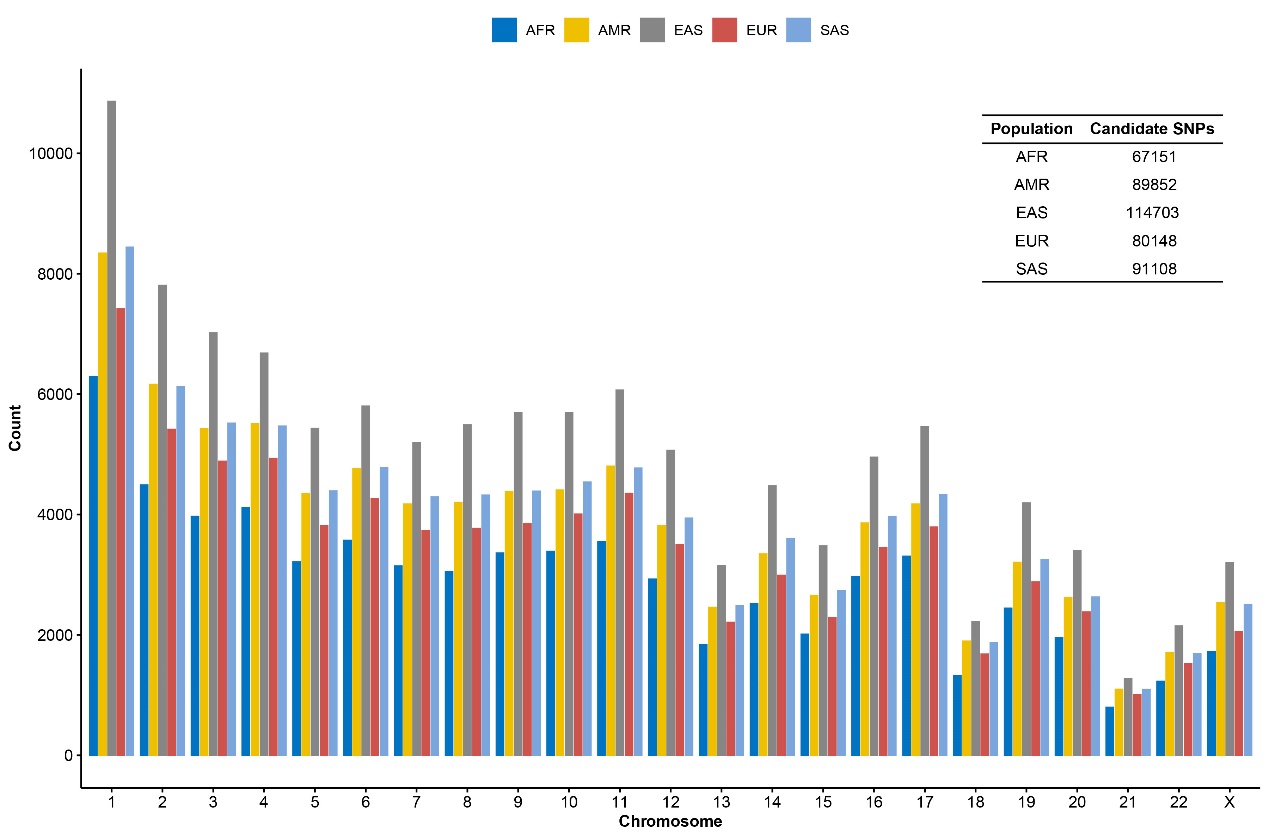


**Fig. S12. Number of suitable SNPs in 1000 Genomes Project populations based on SNP selection criteria in this study.** AFR, African; AMR, Ad Mixed American; EAS, East Asian; EUR, European; SAS, South Asian.


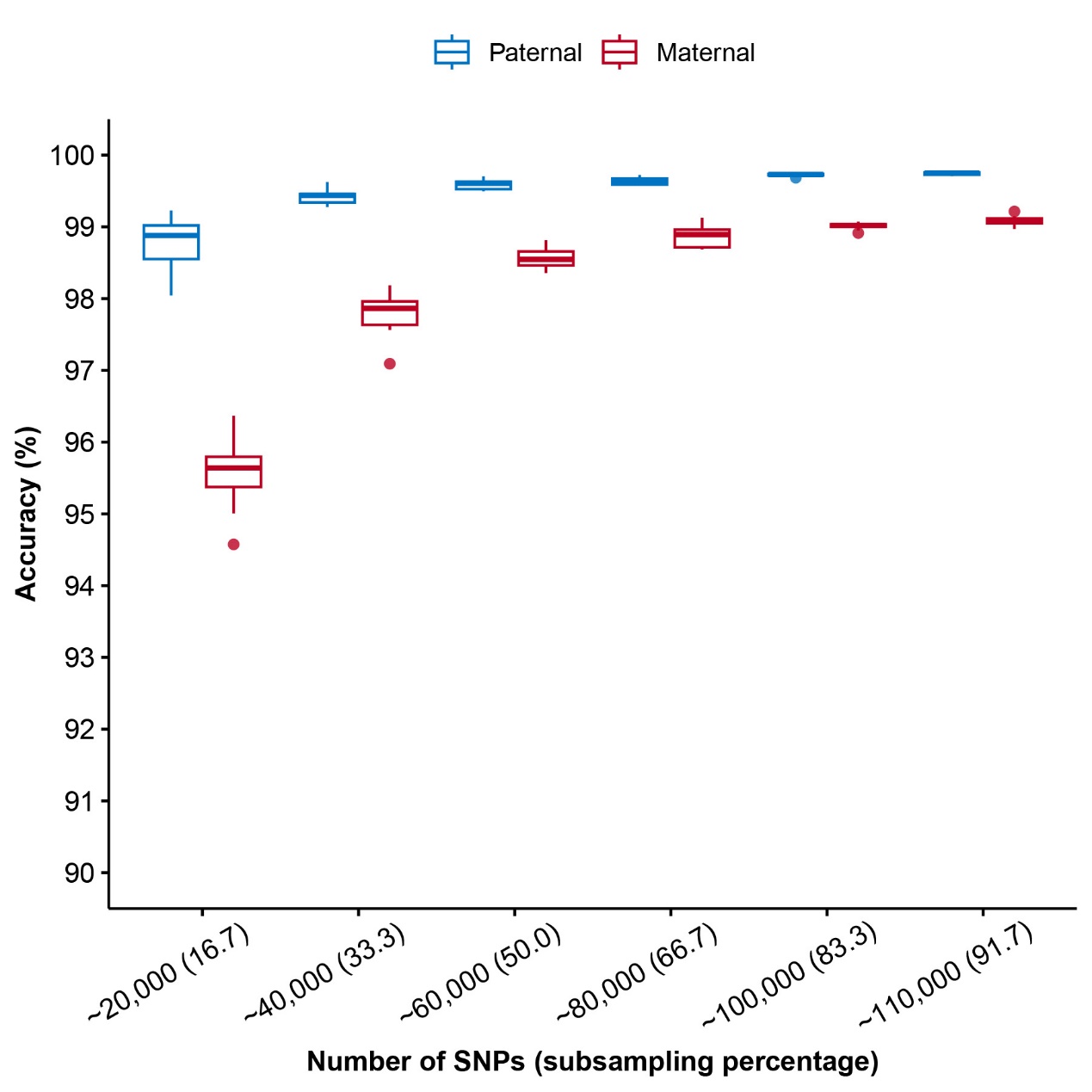


**Fig. S13. Effect of SNP numbers on haplotype accuracy by downsampling simulation.** At a fixed fetal fraction of 10.95%, the x-axis indicates the number of SNPs included, and the y-axis shows the corresponding paternal and maternal haplotype accuracy.
