# Supplementary material for: Universal noninvasive prenatal diagnosis for monogenic disorders using cell-free plasma DNA
Source: Genome Med. 2025 Dec 4;18:4. doi: 10.1186/s13073-025-01588-5 (PMC12797552; doi:10.1186/s13073-025-01588-5)
Supplement: Supplementary file 3 — Additional file 3: Supplementary Methods. [file 13073_2025_1588_MOESM3_ESM.docx]

**Supplementary Methods**

**Parental Haplotypes Construction**

Parental haplotypes were phased based on Mendelian inheritance principles using informative single nucleotide polymorphisms (iSNPs) to discriminate between parental alleles and track their transmission to offspring. iSNPs were defined as loci heterozygous in one parent and homozygous in the other (Additional file 2: Tables S2 and S3; Group 3 for paternal and Group 4 for maternal). The disease-associated haplotype was designated Hap0 (F0/M0) and the unaffected haplotype Hap1 (F1/M1). For example, if the father was heterozygous (AB) and the mother homozygous (AA), the locus was classified as paternal-informative. If the affected proband was homozygous (AA), the paternal Hap0 allele at that locus was inferred to be “A,” consistent with Mendelian segregation. Conversely, if the proband was heterozygous (AB), the Hap0 allele was assigned as “B.” By evaluating a series of such loci, the paternal haplotype could be unambiguously resolved within the target region.

Maternal haplotypes were refined using Group 6 SNPs, defined as loci heterozygous in both parents but homozygous in the proband or an informative relative (Additional file 2: Tables S2 and S3). Once the paternal haplotype inherited by the fetus was determined, Group 6 SNPs within the corresponding paternal haplotype block were treated as homozygous and reclassified as Group 4 SNPs. These reclassified SNPs, together with the original Group 4 set, were then integrated to reconstruct maternal haplotypes.

**Hidden Markov Model (HMM) model approach for fetal haplotype deduction**

Fetal haplotypes were inferred using HMM that incorporated the phased parental haplotypes and the observed allele depth distributions in maternal plasma. Let *N* denote the total number of informative SNPs on the target chromosome, and *S* = {N_j_} represent the observed states, where each Nⱼ contains information of chromosomal position, reference/alternative allele read counts, and phased parental haplotypes at SNP locus j (j = 1, 2, 3, …, N). The hidden states were defined as *Q* = {Hap0, Hap1}, representing the parental haplotype inherited by the fetus. The initial state distribution for each locus was set to {½, ½}.

The emission probability matrix is represented by *B* = {bᵢⱼ}, in which b_ij_ = *P*{Hap i |N_j_}, i ∈ {0,1}, and j = 1, 2, 3, …, N. The probability of Hap i-rich SNP transmitted to the fetus at given site j was calculated as:

$$P\left\{ Hapi | N_{j} \right\}= \frac{P\{N_{j}|Hapi\}}{P\{N_{j}|Hap0\}+ P\{N_{j}|Hap1\}}$$

where $P\{N_{j}|Hapi\}$ is the probability of the observed state *N_j_* at SNP loci j in maternal plasma when Hap *i* was transmitted to the fetus, calculating based on read counts of reference and alternative alleles and the expected proportion under the estimated fetal fraction using a binomial distribution. The transition probabilities matrix was denoted as *A* = {*a_j_*}, where

$a_{j}= \left\{ \begin{matrix} 1-P_{j} & P_{j} \\ P_{j} & 1-P_{j} \end{matrix} \right\}$.

Here, *P_j_* is the probability of recombination between two neighboring SNPs, determined by the genetic distance derived from the 1000 Genomes Project data.
